# Peer Review of “The Order in Speech Disorder: A Scoping Review of State of the Art Machine Learning Methods for Clinical Speech Classification (Preprint)”

**DOI:** 10.2196/76836

**Published:** 2025-05-12

**Authors:** Vanessa Fairhurst, Sylvester Sakilay, Randa Salah Gomaa Mahmoud, Shailee Rasania, J Moonga, Toba Isaac Olatoye, Rameshwari Prasad, Prasakthi Venkatesan, Vasco Medeiros, Uday Kumar Chalwadi

**Affiliations:** 1 PREreview, Portland, OR, United States; 2 Zagazig University, Zagazig, Egypt; 3 King’s College London, London, United Kingdom; 4 Kwara State Teaching Service Commission, Ilorin, Nigeria; 5 Shelby County Health Department, Memphis, TN, United States; 6 Louisiana State University Health Sciences Center Shreveport, Shreveport, LA, United States

**Keywords:** scoping review, machine learning, speech patterns, diagnosis, speech disorders, mental disorders, neurological disorders

*This is the peer-review report for the preprint “The Order in Speech Disorder: A Scoping Review of State of the Art Machine Learning Methods for Clinical Speech Classification.”*

This review is the result of a virtual collaborative live review discussion organized and hosted by PREreview and JMIR Publications on April 10, 2025. The discussion was joined by 29 people: 3 facilitators from the PREreview team, 1 member of the JMIR Publications team, and 25 live review participants, 4 of whom joined as listeners and did not contribute to the review. The authors of this review have dedicated additional asynchronous time after the call over the course of 2 weeks to help compose this final report using the notes from the live review. We thank all participants who contributed to the discussion and made it possible for us to provide feedback on this preprint.

## Summary

Speech is a cornerstone of human communication, intricately connected to our cognitive, neurological, and psychological processes. Speech patterns have emerged as potential diagnostic markers for conditions with varying etiologies. This scoping review [1] elucidates how machine learning (ML) can utilize speech patterns as noninvasive diagnostic biomarkers for neurological, laryngeal, and mental health etiologies. Based on specific inclusion and exclusion criteria that involved a wide spectrum of conditions, ranging from voice pathologies to mental and neurological disorders, the 564 articles compiled in this investigation were condensed to 91. Methods of speech classification were then assessed between 0‐10 based on the diagnostic accuracy of different ML models. High accuracies were reported for Parkinson disease, laryngeal disorders, and dysarthria, whereas disorders like depression, schizophrenia, mild cognitive impairment, and Alzheimer disease (AD) showed promise yet were less consistent. This review emphasizes the need for speech analysis in conditions like obsessive-compulsive disorder and autism, where graded clinical diagnoses are less robust, relative to other disorders. Key strengths of the preprint include its comprehensive coverage of disorders and the current relevance of the literature (post 2016). However, noted limitations include a lack of cross-linguistic model generalizations, a limited coverage of pediatric populations, and sociocultural variations in speech. Despite some ambiguity present in the methodologies, the paper effectively bridges the fields of speech science, artificial intelligence (AI), and clinical diagnostics. Moreover, it highlights the transformative potential of ML in developing personalized scalable diagnostic models while also considering ethical implications, clinical acceptance, and real-world applications.

## List of Major Concerns and Feedback

With “major concerns,” we refer to concerns that the reviewers believe should be prioritized in being addressed in order to ensure the soundness of the study.

Below, we summarize major concerns raised by the live review participants, and whenever possible, we offer suggestions on how to address them.

1. A lack of model validation: More clarity should be provided to highlight the distinction between disease state/features and symptoms. For example, neurodegenerative diseases such as AD and Huntington disease have features similar to neuropsychiatric diseases—schizophrenia, depression, etc. While the symptoms and manifestations can overlap, they are not the same thing; they differ in etiology and characteristics. The failure to delineate those characteristics weakens the study’s overarching question and rationale from the start.
2. A scoping review is meant to provide a wide scope of the literature to map out data and synthesize findings for interpretation and appraisal. There is a major weakness in the findings presented in the tables. At present, the evidence provided does not sufficiently reflect the body of empirical evidence that is available in neurodegeneration, linguistics, and ML methods to achieve the goals in the study aims/objectives. To increase the strength of the analysis and improve the data disseminated in the tables, one option could be to combine the similarities in findings in each table. This task can also improve the presentation of the data in each table.
3. It is not clear why the search is restricted to the PubMed application programming interface and does not include other platforms such as MEDLINE (OVID), Embase (Elsevier), PsycINFO (OVID), CINAHL, Google Scholar, and Web of Science.
4. The methods and results should be reported in accordance with scoping review guidelines, such as PRISMA-ScR (Preferred Reporting Items for Systematic Reviews and Meta-Analyses Extension for Scoping Reviews) [2].
5. The keywords identified to search in databases should be mentioned (it could be added as a supplementary file).
6. The time range of the search was not mentioned.
7. There is a lack of clarity between the neurodegenerative diseases and neuropsychiatric diseases; for example, AD and schizophrenia should be distinguished since AD progresses at various stages that do not necessarily resemble the features of schizophrenia.
8. The dataset size and ratio of healthy controls versus patients are important factors that are necessary to mention in Tables 1-3.
9. Clinical relevance: There is a need to review the profile/demographics of cohorts and groups of participants in the selected studies. This would help to demonstrate the time-course of disease/condition in their application to ML and the nature of the pool of data extracted in the analytical phase of the study (ie, data synthesis and interpretation). That is critical information that could be obtained in the data extraction stage (per PRISMA guidelines). By establishing the clinical relevance here, the paper can better argue how ML methods can help clinical speech classification in neurological and psychiatric diseases for diagnostic purposes.
10. In the inclusion criteria, articles published in English were mentioned, but non-English articles were also included in the study. An explanation for the inclusion of non-English articles was not provided by the authors. Additionally, the study deliberately focused on speech parameters, excluding the analysis of language content, which could provide a more holistic understanding of communicative aspects related to health conditions. Mentioned in 4.6.
11. False negatives: In evaluations, speech can appear healthy even if an individual has a serious health condition, making false negatives an important consideration. Speech-based diagnostics should be an addition to other diagnostic methods, not a stand-alone solution. Authors mentioned this in 4.7.3 as a limitation, but no such attempt was observed in the inclusion of related literature.
12. The authors effectively address key issues such as patient data privacy, informed consent, General Data Protection Regulation (GDPR) compliance, and clinical deployment risks associated with AI-driven speech diagnostics. The inclusion of synthetic speech data as a means to mitigate privacy concerns is a noteworthy strength. To enhance this section, we recommend incorporating specific frameworks or strategies—such as data anonymization, algorithmic transparency, and regulatory guidance—to provide a more robust and actionable ethical foundation for clinical implementation. Ethical considerations, especially around AI deployment, patient data privacy, and consent, should be discussed in more detail.
13. The manuscript provides valuable insights but would benefit from a more comprehensive discussion of its limitations. Key areas that remain unaddressed include the lack of cross-linguistic generalizability of ML models, limited representation of pediatric populations, and sociocultural variations in speech, which may affect the robustness and applicability of the findings. Additionally, issues such as data scarcity, inconsistent data quality, risks of model overfitting, and potential gender bias pose challenges to the development of unbiased and reliable diagnostic tools. The generalization of findings to a broader range of mental health disorders is also a concern; while Parkinson disease and schizophrenia are discussed, the exclusion of numerous other conditions limits the scope of applicability. Clarification on whether these findings can be extended to non–speech-related disorders or a recommendation for future research in this area would strengthen the manuscript.

## List of Minor Concerns and Feedback

### Concerns With Techniques/Analyses

- The manuscript does not thoroughly discuss model validation practices or the potential risk of bias, such as overfitting and limited sample diversity. Although the interpretations are generally sound, a more critical evaluation of the limitations of the individual studies could be included. The authors may wish to include a subsection that summarizes the validation methods used by the reviewed studies.
- There is a lack of standardization in the techniques used across the 91 studies, as most studies employ different speech tasks, which may impact the biomarkers activated or identified. Additionally, speech impairment changes with disease progression, so it would be useful to include age and more information about the disease state.
- The References section shows inconsistencies in formatting and needs to be revised to follow a uniform citation style in accordance with a journal’s guidelines.
- The number of included articles is stated as 91, but Tables 1-3 present only 77 studies, while Table 4 shows 64. This discrepancy is unclear and may confuse readers. Kindly provide an explanation for the differences in the number of articles across the tables. You can include a brief footnote in the manuscript on why those articles were excluded.
- In section 2.6 “Articles Found,” it is unclear why articles including magnetic resonance imaging, computed tomography, electroencephalogram, image, wearable sensors, video, transcription, or multimodal data were excluded. Clarify the specific scope and focus of the review that justified the exclusion of these factors.
- The year of publication listed in the table looks disorganized. The authors could reorder the studies in the table in either ascending or descending order of year of publication to help readers identify the progression of research over time.
- Please clarify why GPT-4 or GPT-4.5 (instead of GPT-3.5) was not used despite being available at the time of the study.
- Under “3. Results,” the authors could use more clarifying language while describing languages used (English was the most common language, but the results also included studies on Chinese, Greek, Spanish, Malay, and Hebrew). Since non-English language studies were excluded. It looks like they may have used studies where test sets were in different languages. Suggestion: The sentence under “3. Results” can be restructured to clarify the same.

### Details for the Reproducibility of the Study

- The reproducibility of the Grading of Recommendations Assessment, Development and Evaluation (GRADE) scoring is limited due to the absence of a clearly defined rubric or framework. Provide a detailed explanation or scoring rubric highlighting how each criterion of the GRADE scoring system was applied.
- An insufficient search strategy will make it difficult for other researchers to replicate or validate the review process. Authors should expand the Method section and describe the databases used, the search terms, the inclusion or exclusion criteria, and any screening processes like PRISMA flow. This will improve the credibility and reproducibility of this study.

### Figures and Tables

- Some captions lack the specific details of the dataset used, the method languages, and the clinical settings. Also, some tables are overly dense. Revise these captions to include contexts like data sources, methodology, and clinical backgrounds. The authors may consider breaking dense tables into subcategories to enhance clarity.
- The reference numbers are missing in the first column of all tables and should be added in brackets following the author names (eg, “Alan et al [23]”) to allow quick cross-referencing with the reference list.
- Not all the tables were cited within the main text of the article.
- The description of Figure 1 should be expanded further. Moreover, the authors should put the name of the primary author before the reference and the year of publication (eg, “(NAME et al (2XXX) [114]”). Figure 1 should also be revised to increase its readability. Perhaps, the authors could minimize the quadrants and increase the size of the text font.
- Divide the Participants column in Tables 1-3 into “Target Patients” and “Control Patients” to improve readability.
- It would be helpful if the tables listed the time duration of the studies.
- There are multiple spelling mistakes and excessive use of undefined abbreviations, especially in tables. There is also a lack of standardization in reporting speech features and methods, making comparison difficult.
- Could combine similar findings in each table (ie, combine cells), but keep authors’ citations in the tables.

### Additional Comments

- The manuscript would benefit from figures, diagrams, or charts that summarize key trends such as ML model performance across various disorders, as well as a visual overview of the review process.
- There is insufficient detail on why speech disorders were chosen as the focal point in a rapidly expanding domain of ML-based diagnostics. Authors should add content and references to emphasize the broader relevance of ML in diagnostics and explain the reason behind their narrowing the scope to speech-based disorders.
- Number the references in order, starting with “#41.”
- In both the Abstract and Results sections, please write the abbreviation “OCD” as “obsessive-compulsive disorder (OCD).”
- In the Rationale and Results section, please revise the sentence “ML provides enables” by removing one of the verbs to correct the grammar.
- Please add a reference to the GRADE rating.
- In the Dysarthria, general section: please identify the abbreviation “PWSI-AI-AC” as “patch-wise wave splitting and integrating AI system for audio classification.”
- In the “Alzheimer’s Disease (AD)” section, please identify the abbreviation “eGeMAPS” as the “extended Geneva Minimalistic Acoustic Parameter Set.”
- “Gomez et al” should be corrected to “G´omez-Rodellar et al” in the “Parkinson’s Disease (PD)” section and Table 1.
- In the “Incorporating ML Based Speech Assessment in Clinical Practice” section, please identify “GDPR” as “General Data Protection Regulation.”
- In the Methods section, the phrase “focused on Parkinson, [3] focused on psychiatric disorders, and [4] focused on depression and suicide risk” should be revised to “focused on Parkinson, [3] on psychiatric disorders [4], and on depression and suicide risk.”
- The title includes “state of the art,” which may be misleading as the GPT-3.5-turbo model was used in this paper, and since February 27, 2025, the most current version, GPT-4.5 model, has been released. Authors should specify the model type in the title.
- Acronyms such as “CNN” and “AUC” are used without definition on page 6.
- “3.2.6 Reinke’s edemba”: It should be “edema” not “edemba.”
- This manuscript requires comprehensive proofreading and editing.

We thank the authors of the preprint for posting their work openly for feedback. We also thank all participants of the live review call for their time and for engaging in the lively discussion that generated this review.

## Abbreviations

AD: Alzheimer disease
AI: artificial intelligence
GDPR: General Data Protection Regulation
GRADE: Grading of Recommendations Assessment, Development and Evaluation
ML: machine learning
PRIMSA-ScR: Preferred Reporting Items for Systematic Reviews and Meta-Analyses Extension for Scoping Reviews

## References

[1] Moell B, Aronsson FS, Östberg P, Beskow J (2025). The order in speech disorder: a scoping review of state of the art machine learning methods for clinical speech classification. arXiv.

[2] Tricco AC, Lillie E, Zarin W (2018). PRISMA Extension for Scoping Reviews (PRISMA-ScR): checklist and explanation. Ann Intern Med.

[3] Low DM, Bentley KH, Ghosh SS (2020). Automated assessment of psychiatric disorders using speech: a systematic review. Laryngoscope Investig Otolaryngol.

[4] Cummins N, Scherer S, Krajewski J, Schnieder S, Epps J, Quatieri TF (2015). A review of depression and suicide risk assessment using speech analysis. Speech Commun.

